# Combining Innovative Bioink and Low Cell Density for the Production of 3D-Bioprinted Cartilage Substitutes: A Pilot Study

**DOI:** 10.1155/2020/2487072

**Published:** 2020-01-21

**Authors:** Christel Henrionnet, Léa Pourchet, Paul Neybecker, Océane Messaoudi, Pierre Gillet, Damien Loeuille, Didier Mainard, Christophe Marquette, Astrid Pinzano

**Affiliations:** ^1^UMR 7365 CNRS-Université de Lorraine IMoPA, Biopôle de l'Université de Lorraine, Campus Brabois-Santé, 9, Avenue de la Forêt de Haye, BP20199, 54505 Vandœuvre-Lès-Nancy, France; ^2^Platform 3D Fab, University of Lyon, CNRS, INSA, CPE-Lyon, ICBMS, UMR 5246, 43, Bd du 11 Novembre 1918, F69622 Villeurbanne Cedex, France; ^3^Laboratoire de Pharmacologie, Toxicologie et Pharmacovigilance, Bâtiment de Biologie Médicale et de Biopathologie, CHRU de Nancy-Brabois, 5 Rue du Morvan, F54511 Vandœuvre-lès-Nancy, France; ^4^Service de Rhumatologie, CHRU de Nancy-Brabois, Bâtiment des Spécialités Médicales, 5 Rue du Morvan, F54511 Vandœuvre-lès-Nancy, France; ^5^Service de Chirurgie Orthopédique, Traumatologique et Arthroscopique, Hôpital Central, CHRU of Nancy, F54035 Nancy, France; ^6^Contrat d'Interface, Service de Rhumatologie, CHRU de Nancy-Brabois, Bâtiment Spécialités Médicales, F54511 Vandœuvre-lès-Nancy, France

## Abstract

3D bioprinting offers interesting opportunities for 3D tissue printing by providing living cells with appropriate scaffolds with a dedicated structure. Biological advances in bioinks are currently promising for cell encapsulation, particularly that of mesenchymal stem cells (MSCs). We present herein the development of cartilage implants by 3D bioprinting that deliver MSCs encapsulated in an original bioink at low concentration. 3D-bioprinted constructs (10 × 10 × 4 mm) were printed using alginate/gelatin/fibrinogen bioink mixed with human bone marrow MSCs. The influence of the bioprinting process and chondrogenic differentiation on MSC metabolism, gene profiles, and extracellular matrix (ECM) production at two different MSC concentrations (1 million or 2 million cells/mL) was assessed on day 28 (D28) by using MTT tests, real-time RT-PCR, and histology and immunohistochemistry, respectively. Then, the effect of the environment (growth factors such as TGF-*β*1/3 and/or BMP2 and oxygen tension) on chondrogenicity was evaluated at a 1 M cell/mL concentration on D28 and D56 by measuring mitochondrial activity, chondrogenic gene expression, and the quality of cartilaginous matrix synthesis. We confirmed the safety of bioextrusion and gelation at concentrations of 1 million and 2 million MSC/mL in terms of cellular metabolism. The chondrogenic effect of TGF-*β*1 was verified within the substitute on D28 by measuring chondrogenic gene expression and ECM synthesis (glycosaminoglycans and type II collagen) on D28. The 1 M concentration represented the best compromise. We then evaluated the influence of various environmental factors on the substitutes on D28 (differentiation) and D56 (synthesis). Chondrogenic gene expression was maximal on D28 under the influence of TGF-*β*1 or TGF-*β*3 either alone or in combination with BMP-2. Hypoxia suppressed the expression of hypertrophic and osteogenic genes. ECM synthesis was maximal on D56 for both glycosaminoglycans and type II collagen, particularly in the presence of a combination of TGF-*β*1 and BMP-2. Continuous hypoxia did not influence matrix synthesis but significantly reduced the appearance of microcalcifications within the extracellular matrix. The described strategy is very promising for 3D bioprinting by the bioextrusion of an original bioink containing a low concentration of MSCs followed by the culture of the substitutes in hypoxic conditions under the combined influence of TGF-*β*1 and BMP-2.

## 1. Introduction

Sports trauma and overuse largely contribute to the occurrence of chondral lesions in weight-bearing areas of young patients. Cartilage defects are very common lesions and are reported in 63% of patients who undergo arthroscopy [1]. Cartilage is a stratified avascular tissue with very limited repair capabilities. Its regeneration would make possible to repair hyaline cartilage and thereby reduce its degeneration in order to prevent the development of osteoarthritis.

The clinical surgical reference treatment for focal chondral lesions remains the mosaicplasty, which uses osteochondral biopsies harvested from a non-weight-bearing area on the periphery of the same joint that is being repaired. The results of mosaicplasty are relatively satisfactory for the first 2 years but experience a steep failure rate over the next 2 years. A high failure rate is commonly recorded (approximately 55%) [2], and collecting osteochondral plugs from the knee joint often results in considerable donor-site morbidity for knee-to-knee (6%) and knee-to-ankle (17%) transplants after mosaicplasty procedures [3].

Currently, cartilage repair strategies are mainly focused on tissue engineering [4], which consists of creating a functionalized material that mimics the native tissue. To this end, 3D bioprinting is a rapidly emerging technique that uses the simultaneous 3D deposition of living cells inside supportive dedicated biocompatible biomaterials [5]. This technique permits to obtain a well-defined, often complex, form of custom-made dimensions to be obtained using a layer-by-layer biofabrication strategy. For cartilage engineering, the most commonly used 3D printing process is the extrusion-based bioprinting, which is well known to be able to generate viable constructs several centimeters in size [6, 7].

Most of the published 3D bioprinting studies have utilized high cellular density, which is not representative of the native cartilage. In fact, chondrocytes only represent 2% of hyaline cartilage volume [8]. Concerning these cell candidates, chondrocytes were initially studied as “magic bullets” for cartilage engineering. However, both their poor availability within cartilage and their fibroblastic dedifferentiation during the monolayer expansion phase remain crucial restricting factors. With this in mind, researchers now use mesenchymal stem cells (MSCs) as a powerful alternative: they are sufficiently robust to survive the shear stress and pressure inherent to the bioprinting process [9] and exert a good proliferation capacity and an excellent potential for TGF-*β*-driven chondrogenicity in a purpose-made dedicated 3D environment [10]. MSCs can be extracted from multiple tissues, such as bone marrow, adipose tissue, synovium, periosteum, and muscle, and are capable of renewing themselves through cell division and can differentiate into multilineage cells, including articular cells [11], with the typical ancillary chondral extracellular matrix production of type 2 collagen and proteoglycans.

The optimization of the 3D bioprinting process (i.e., extrusion, droplet, or laser [12]) and the formulation of a bioink with a good printability are key to producing 3D cartilage substitutes several centimeters in size containing living cells [13, 14]. The extrusion-based bioprinting (EBB) process is the most suitable technique for the requirement of cartilage tissue engineering. Combining decellularized extracellular matrix in bioink and MSCs allows the production of 3D tissues by a deposition process, with custom-designed layers [15–19]. The bioink used for cartilage substitutes is generally a hydrogel which facilitates homogeneous cell encapsulation and allows a sufficiently resistant 3D structure. It is a promising one for cartilage engineering and regenerative medicine application due to the balance of biochemical and physical characteristics [20–22].

Natural hydrogels have been extensively used for cartilage engineering and bioink formulations for EBB. Alginate is a low-cost biomaterial extracted from brown algae with good printability and excellent biocompatibility [12, 23, 24]. Gelatin, an abundant and inexpensive material extracted from denatured collagen in animal skin and bones, displays a reversible thermosensitive gelation mechanism and exerts a lower antigenicity and a better biocompatibility compared to collagen [12]. Fibrinogen is a glycoprotein that forms fibrin through a proteolytic reaction with thrombin, thus allowing a rapid gelation to maintain the 3D shape of the 3D-printed constructs [25]. Based on these three biomaterials, Pourchet et al. [26] recently developed a composite bioink that guarantees cytocompatibility during 3D bioprinting and cell proliferation after bioprinting and enables the production of full-thickness skin tissues with a low cell concentration (1 M cells/mL) contrasting with those previously used for cartilage engineering (4 M to 50 M/mL) [27].

In the present pilot study, the bioink developed herein was validated and applied to the *in vitro* production of tissue-engineered cartilage substitutes for the regenerative therapy of chondral focal lesions. Our approach takes advantage of using a low MSC density, which is more representative of native cartilage. Although MSCs are not often used in cartilage bioprinting [28–32], they are very promising for cartilage engineering because of their chondrogenic potential and their excellent availability (e.g., from the bone marrow) for autologous or allogeneic grafts. To this end, we first evaluated the biocompatibility of the bioink and the effect of the 3D bioextrusion process on MSC metabolism and their genic expression profile and ECM production, at two different cell concentrations, that mimicked the cell density of native cartilage. We then determined the best differentiation/maturation conditions (in terms of growth factors and hypoxic stress) for the MSC-driven chondrogenic differentiation of cartilaginous substitutes (Suppl1).

## 2. Material and Methods

### 2.1. Stem Cell Isolation and In Vitro Expansion

Mesenchymal stem cells (MSCs) were isolated from human bone marrow following total hip arthroplasty (for advanced osteoarthritis (OA), grade 3-4 Kellgren-Lawrence staging, patients aged 60-80 years) after informed consent and with the approval of the local ethical committee (File DC 2014—2148, authorized 2014, July, 10^th^). To this end, heparinized bone marrow was diluted in PBS (phosphate-buffered saline, pH 7.4) solution and then centrifuged at 1600 rpm for 5 min. The pellets were diluted in culture medium and then were seeded in 100 mm in diameter Petri dishes at 4 × 10^6^ cells/dish at 37°C in a humidified atmosphere containing 5% (*v*/*v*) CO_2_. The medium was not changed for the first 3 days. The nonadherent cells were removed during sequential media changes.

During expansion of the monolayers, MSCs were cultured in low glucose level Dulbecco's modified Eagle medium with 1 g/L of glucose (DMEM-LG, 31885, Gibco) supplemented with 10% (*v*/*v*) of fetal bovine serum (FBS, Sigma), 1 ng/mL of bFGF (Miltenyi), 2 mM of glutamine (Gibco), and 1% (*v*/*v*) penicillin streptomycin (Gibco). The medium was changed 2 times per week until cells became confluent. Once 80% of the confluence was attained, the MSCs were trypsinized and plated at a density of 0.5 × 10^6^ cells/flask. During the last passage (P3) and before seeding in hydrogels, a chondrogenic predifferentiation step was performed. To do so, MSCs were cultured with complete differentiation medium [33] composed of DMEM with 4.5 g/L of glucose supplemented with FBS, sodium pyruvate (110 *μ*g/mL, Gibco), bFGF (1 ng/mL, Miltenyi), 1% penicillin streptomycin (Gibco), and the chondrogenic supplements: proline (40 *μ*g/mL, Sigma), L-ascorbic acid-2-phosphate (50 *μ*g/mL, Sigma), and dexamethasone (0.1 *μ*M, Sigma). MSCs could be expanded in monolayer until passage 5 without loss of their undifferentiated phenotype and without haematopoietic cell contamination [34]. Three passages are required to obtain comparable >90% purity [35].

### 2.2. 3D Bioprinting of Engineered Cartilage Substitutes

The chosen printer was developed by the authors and is fully compatible with laboratory safety standards [26]. The bioprinting patterns (GCode) were generated using Repetier Host Software® (Hot-World GmbH & Co. KG, Knickelsdorf, 4247877 Willich Germany) in order to generate a rectangular shape cartilage substitute that was 1 cm in length, 1 cm in width, and 4 mm in thickness. The bioink was formulated as a mixture of 10% (*w*/*v*) bovine gelatin (Sigma-Aldrich, France), 1% (*w*/*v*) very low viscosity alginate (molecular weight: 216.121 g/mol; Alfa Aesar, France), and 2% (*w*/*v*) fibrinogen (Sigma-Aldrich, France) at 37°C [26]. The rheological properties of this bioink have already been published [26]. All solutions (fibrinogen, alginate, and gelatin) are prepared in sterile conditions the day before printing. They are placed at 37°C for a good dissolution of the powders. Gelatin is obtained in NaCl (20%). Fibrinogen is prepared in culture medium (160 mg/2 mL), and alginate is prepared in NaCl (4%). The cells are taken up in 2 mL of fibrinogen to which 4 mL of gelatin and 2 mL of alginate are added (total of 8 mL).

Just before bioprinting (D0), human MSCs were trypsinized and seeded in the bioink, after which they were homogenized and loaded in a sterile 10 mL syringe equipped with a 450 *μ*m diameter tronconical bioprinting nozzle. The cells were trypsinized, counted, and resuspended in 2 mL of fibrinogen solution (160 mg in 2 mL culture medium). 4 mL of gelatin (20% in NaCl) and 2 mL of alginate (4% in NaCl) are added. The 8 mL obtained in this way is homogenized using a Microman “special viscous media” pipette, then taken up in a 10 mL syringe. The syringe containing the bioink is then maintained at room temperature for 30 minutes, which is the time required to obtain a bioink whose viscosity is compatible with good quality layer-by-layer printing and to avoid bubbles. This 30-minute time was previously developed by Pourchet et al. in their previous work.

The bioink containing MSCs was bioprinted layer by layer to build the tissue-engineered cartilage substitutes. Following bioprinting, the cartilage substitutes were placed in Petri dishes containing a polymerization solution composed of 4% CaCl_2_ (*w*/*v*) and thrombin (25 U/mL) and were incubated and shaken simultaneously for 1 hour at 37°C in an incubator heating unit (Heidolph® Model 1000). After polymerization and washing with PBS solution, the printed substitutes were cultured in culture media of various compositions. The medium was changed 3 times per week. Photos of the process are given in Suppl2.

### 2.3. Mitochondrial Activity Assay

The mitochondrial activity in the cartilage 3D-bioprinted substitutes was evaluated by MTT (3(4,5-dimethylthiazol-2-yl)-2,5-diphenyltetrazolium bromide) assays at different times for all studied conditions [36]. One hundred microliters of culture medium and 25 *μ*L of MTT solution (5 mg/mL) were added to each well containing a bioprinted substitute, and the plates were incubated in the dark at 37°C in 5% CO_2_ for 3 hours. An intense purple colored formazan derivative formed during active cell metabolism that was eluted and diluted in a solution containing 80 g sodium dodecyl sulfate and 200 mL of dimethylformamide and 200 mL of water (pH 4.7). The absorbance was measured at 580 nm with a spectrophotometer (Multiskan Ex, Thermo Labsystems) on the following time points: on D0 with or without bioextrusion, with or without polymerization, and on D3, D7, D14, D21, and D28.

### 2.4. Real-Time RT-PCR Analysis

The 3D-bioprinted cartilage substitutes were frozen at -80°C until analysis. The RNA extraction was performed using RNeasy Mini Kit (Qiagen), according to the manufacturer's instructions. After extraction, a reverse transcription was performed with 500 ng of RNA by using an Omniscript RT Kit (Qiagen). Real-time polymerase chain reaction (PCR) was performed using QuantiTect™ SYBR Green PCR (Qiagen). The relative quantification was performed using a standard curve generated from a purified PCR product for each tested gene, at concentrations ranging from 10^−3^ to 10^−6^ ng/*μ*L. For the standardization of the gene expression levels, the results were expressed as the ratio of the mRNA level of each gene of interest and that of the *RPS29* gene. This gene (*RPS29*) was referred to as a housekeeping gene, which typically is a constitutive gene that is expressed at relatively constant levels independently of the experimental conditions. The genes examined in this study were those encoding type II collagen (*COL2A1*), type X collagen (*COL10A1*), aggrecan (*ACAN*), versican (*VCAN*), SRY- (sex-determining region Y-) box 9 (*SOX9*), cartilage oligomeric matrix protein (*COMP*), alkaline phosphatase (*ALP*), osteocalcin (*BGLAP*), and osterix (*OSX)*. The sequences and product sizes are presented in Table 1.

### 2.5. Effect of Bioextrusion and the Polymerization Process on Mitochondrial Activity

To study the effect of bioextrusion during the 3D bioprinting process and the influence of polymerization, which is necessary to maintain the substitutes composed of alginate-based bioinks, we designed 2 types of substitutes which were polymerized or not for one hour in 4% (*w*/*v*) CaCl_2_. The first substitute was produced using the 3D bioprinting and the other was produced directly with a culture micropipette. For this latter condition, as it is a nonprinted control, alginate-based bioink is directly put into the Petri dish throughout a culture micropipette (Microman) to mimic the shape and volume of the 3D-printed substitute without using the EBB process. Two concentrations of MSCs in the bioink were tested: 1 million (1 M) and 2 million cells (2 M) per mL of bioink. The mitochondrial activity in the presence of all these substitutes was evaluated immediately after the bioprinting process to assess the respective effects of the bioextrusion and/or polymerization processes.

### 2.6. Influence of the MSC Density in the Bioink on TGF-*β*1-Driven Differentiation

To assess the best cell concentration to be used for the following experiments, 3D-bioprinted cartilage substitutes were cultured either in a minimum medium containing only 1% ITS (ITS+premix, BD Biosciences) or an enriched medium supplemented with TGF-*β*1 (10 ng/mL, Miltenyi) to induce the chondrogenic differentiation of MSCs and cartilaginous matrix synthesis for 28 days. ITS is used in order to avoid fetal bovine serum, which already contains growth factors. The medium was changed 3 times per week during the substitutes' maturation. On D28, the gene expression of the chondrogenic, hypertrophic, and osteogenic markers was analyzed.

### 2.7. Effect of Environment (Growth Factors and Oxygen Tension) on Chondrogenicity

After chondrogenic predifferentiation was induced in the monolayers, the bioink was prepared with MSCs at a predefined concentration of 1 million cells/mL. The 3D-bioprinted cartilage substitutes were produced and cultured without chondrogenic medium containing 1% ITS as a control or with different culture media enriched with the following growth factor combinations: TGF-*β*1 (10 ng/mL), TGF-*β*3 (10 ng/mL), BMP-2 (100 ng/mL), TGF-*β*1 (10 ng/mL)+BMP-2 (100 ng/mL), and TGF-*β*3 (10 ng/mL)+BMP-2 (100 ng/mL); the substitutes were cultured under normoxic (21% O_2_) or hypoxic (5% O_2_) conditions for 28 and 56 days. The medium was changed 3 times per week. At D28 and D56, analyses were performed to determine the mitochondrial activity, gene expression of chondrogenic, hypertrophic, and osteogenic markers, and finally the quality of the cartilaginous matrix synthesis inside the 3D cartilage substitutes using histology and immunohistochemistry.

### 2.8. Histological Evaluation of ECM Synthesized inside 3D-Bioprinted Cartilage Substitutes

The synthesis of cartilaginous ECM was evaluated through histology at D28 and D56. The 3D-bioprinted cartilage substitutes were fixed with 4% paraformaldehyde solution containing in addition 10 mM CaCl_2_ and 0.1 M sodium cacodylate (pH 7.4) for 24 hours at 4°C. Then, the substitutes were washed overnight at 4°C in 0.1 M sodium cacodylate (pH 7.4) containing 50 mM BaCl_2_, dehydrated in ethanol, and embedded in paraffin. Five-micrometer sections were cut and stained using hematoxylin eosin saffron (cell counting and morphology), Alcian blue (pH 1.3) (sulfate glycosaminoglycan (GAG) content visualization), and alizarin red (pH 4.2) (calcium deposition visualization). The histological studies were based on the observation of 4-6 sections for each experimental condition. A single image that is representative of each experimental condition is presented herein.

### 2.9. Immunohistochemistry of Type II Collagen inside 3D-Bioprinted Cartilage Substitutes

Type II collagen was chosen as the characteristic marker of the hyaline cartilage phenotype to assess the degree of chondrogenic MSC differentiation in the 3D-bioprinted cartilage substitutes. Immunohistochemistry analyses were performed with the LSAB®+ kit (HRP, Dako) using anti-type II collagen monoclonal antibodies (Labvision, France). Paraffin-embedded tissues (5 *μ*m) were deparaffinized, treated with pepsin (0.4% *w*/*v*, Sigma) for 30 min at room temperature, and incubated with a hydrogen peroxide blocking solution for 5 min to block the endogenous peroxidases, as precisely described in our previous work [37]. The sections were counterstained with hematoxylin and mounted with resin.

### 2.10. Densitometry of GAGs and Collagen Type II Staining Using ImageJ

For histology and immunohistochemistry, the tissues were imaged by using a DMD 108 optical microscope (Leica®, France) at a 4x magnification and evaluated with ImageJ software (U. S. National Institutes of Health, Bethesda, Maryland, USA). Transmission light images of Alcian blue staining and of collagen type II immunohistochemistry were recorded and evaluated by a semiquantitative method using the image analysis software ImageJ as we previously described [37]. Briefly, the transmitted light images were recorded and evaluated by a semiquantitative custom method to calculate the stained percentage area (Alcian blue stain and immunohistochemical markers of collagen type II). Densitometry analysis was carried out on images of 6 different sections taken at 4x magnification to visualize the entire construction and by two different experimenters with less than 2% error between the observations. For the evaluation of the microcalcifications identified using alizarin red staining, we assessed the mean number of calcium deposits per image at 4x magnification,

### 2.11. Data Analysis

Analyses were performed in triplicate (in 3 to 4 patients) for each experimental condition. Data are then presented as the mean ± standard deviation to depict the intra- and interindividual variations. For the standardization of gene expression levels, the results were expressed as the ratio of the mRNA level of each gene of interest and that of the RPS29 gene at D28 and D56. Significance was determined by a one-way ANOVA comparison with Dunnett's post hoc test to compare each batch with a control experiment (ITS condition). When necessary, the significance of the interaction was assessed with a two-way ANOVA followed by Bonferroni's test to assess the influence of cell concentration or environmental factors for each condition. Three-way ANOVA was finally performed to simultaneously assess the respective influences of growth factors, oximetry, and the time points. The details are provided in each legend. The statistical analyses were performed with GraphPad Prism®V8.

## 3. Results

### 3.1. Effects of the Bioextrusion Process, Polymerization Steps, and Cell Concentrations

**Figure 1:**
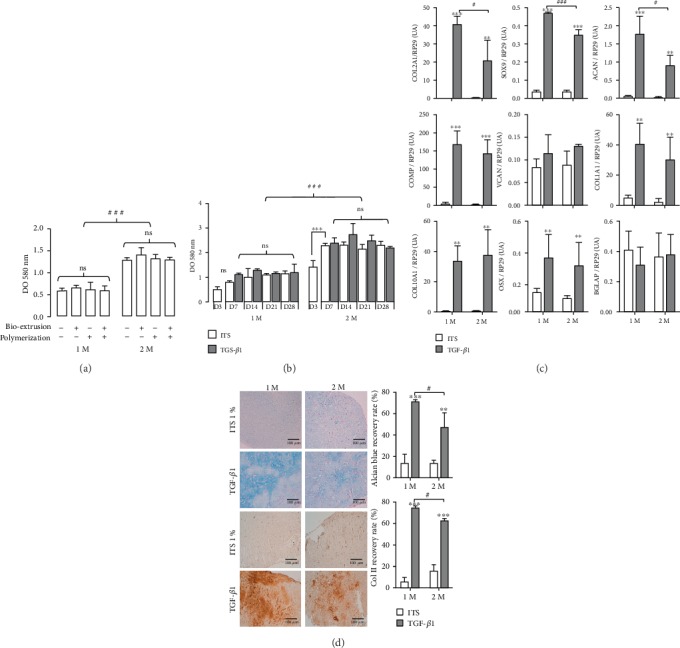
Respective influence of the bioprinting process and chondrogenic differentiation on MSC metabolism, gene profiles, and MEC production at two different MSC concentrations. (a) MSC mitochondrial activity in substitutes with two different cell concentrations (1 million or 2 million cells/mL; 1 M or 2 M, respectively) with or without extrusion-based 3D bioprinting and/or cross-linking. Data are presented as the mean ± SD. The experiments were carried out in triplicate. One-way ANOVA did not show any significant differences between each group and the respective control and between each condition (ns: not significant), but two-way ANOVA followed by a Bonferroni post hoc test demonstrated a significant correlation with the cell concentration (^###^*p* < 0.001). (b) MSC mitochondrial activity in substitutes with two different cell concentrations (1 M or 2 M during TGF-*β*1-driven chondrogenesis). Cell differentiation was induced by TGF-*β*1 exposure on D3. MTT assays were performed on D7, D14, D21, and D28. Data are presented as the mean ± SD. The experiments were carried out in triplicate. For 1 M and 2 M, there was no significant difference at each time point studied in comparison with ITS conditions (Dunnett's test). In contrast, two-way ANOVA with a Bonferroni post hoc test demonstrated a significant correlation with the cell concentration. (c) Chondrogenic, fibrotic, and osteogenic gene expression (TGF-*β*1 versus ITS conditions) in 3D-printed substitutes. Real-time qPCR was performed on D28 for the two cell concentrations (1 million (1 M) or 2 million (2 M) cells/mL). Data are presented as the mean ± SD. The experiments were carried out in triplicate. In the first step, each TGF-*β*1 condition was compared with its ITS control with Student's *t*-test: ^∗^*p* < 0.05, ^∗∗^*p* < 0.01, and ^∗∗∗^*p* < 0.001. Then, two-way ANOVA assessed the global influence of cell concentration in both media with Bonferroni's test. ^#^*p* < 0.05, ^###^*p* < 0.001; this means that in these conditions, 1 M induced significantly increased chondrogenic gene expression in the presence of TGF-*β*1 than 2 M. (d) Histological and immunohistochemical analyses of 3D-printed substitutes seeded with MSCs (1 M or 2 M) in both culture conditions (1% ITS or TGF-*β*1) on D28. The proteoglycans were observed by Alcian blue staining and type II collagen was observed using immunohistochemistry (in red). Quantitative analysis of the histological staining (scale bar 100 *μ*m) with Alcian blue and the immunohistochemical evidence of type II collagen in 3D-printed cartilage substitutes was performed with ImageJ. The results are expressed as the percentage mean ± SD of the positively stained area (4 experiments). In the first step, each TGF-*β*1 condition was compared with its ITS control with Student's *t*-test: ^∗∗∗^*p* < 0.001. Then, a two-way ANOVA followed by Bonferroni's post hoc test assessed the global influence of cell concentration in both media. ^#^*p* < 0.05, which means that in these conditions, 1 M induced significantly greater staining of TGF-*β*1 than 2 M. DO: absorbance.

The effects of the bioextrusion process and the bioink polymerization step on mitochondrial cell activity were assessed by MTT assays of the substitutes produced through bioprinting or manually. Both substitute types were then separated into 2 groups according to whether they were generated with or without the 1-hour polymerization step in CaCl_2_. The results are shown in Figure 1(a). No effect of bioextrusion was observed, regardless of the cell density. Similarly, no difference was noticed between the polymerized and unpolymerized 3D-bioprinted cartilage substitutes, regardless of the seeding density (1 million/mL or 2 million/mL). Moreover, it appeared that the measured mitochondrial activity was directly proportional to the density of the cells seeded in the bioink (Figure 1(a)). These results were confirmed by a DNA assay showing a 2-fold greater amount of DNA at a density of 2 M compared to that at 1 M on D3 (Suppl3).

### 3.2. Effect of the Cellular Density on TGF-*β*1-Driven MSC Differentiation

#### 3.2.1. Mitochondrial Activity

On D3, the results showed a difference in the overall mitochondrial activity in the substitutes containing 1 M or 2 M cells/mL (Figure 1(b)). In fact, for the substitute containing 2 M cells/mL, the absorbance instead DO at 580 nm was 2-fold greater than that for the substitute containing 1 M cells/mL, which perfectly correlated with the initial number of cells. On D7, we observed a slight equivalent increase in mitochondrial activity at both cell concentrations that was inherent to the cell proliferation within the substitutes in the first few days. After D7, the mitochondrial activity remained stable until D28 at both cell concentrations without a significant difference between the two culture conditions (ITS or TGF-*β*1) as proliferation decreased and differentiation increased (Figure 1(b)).

#### 3.2.2. Gene Expression

The study of chondrogenic gene expression was performed with the bioprinted substitutes after 28 days of culture either in a minimum medium containing 1% ITS or in medium enriched with TGF-*β*1, which served as a chondrogenic inducer. The results are presented in Figure 1(c). At each density (1 M or 2 M), the effect of TGF-*β*1 was compared to the effect of ITS. As predicted, TGF-*β*1 induced the typical chondrogenic differentiation of MSCs seeded in the 3D-bioprinted substitutes with the significant overexpression of *COL2A1*, *COL10A1*, *ACAN*, *SOX9*, and *COMP*. In contrast, TGF-*β*1 supplementation induced no significant overexpression of an osteogenic marker such as *BGLAP* (osteocalcin). Among the fibrotic markers, only *COL1A1* was significantly overexpressed, while *VCAN* expression remained stable. In addition, it is worth noting that the expression of typical chondrogenic genes (namely, *COL2A1*, *ACAN*, and *SOX9*) was more enhanced within the 1 M substitute.

#### 3.2.3. Histology and Immunohistochemistry

The analysis of the 3D-bioprinted cartilage substitutes at D28 demonstrated first that a dense layered structure can be maintained for at least 4 weeks without altering the resident cells. Indeed, the HES staining (Suppl 4) did not reveal any alteration of the cells regardless of the culture conditions (no cell death). Moreover, a homogeneous cell distribution and a lack of cell mortality (purple color) were observed, and there was no obvious difference in cell density between the 1 M and 2 M conditions.

The cartilage substitutes obtained with ITS only were characterized by a low level of synthesis and poor-quality ECM, and there was no significant difference between the 1 M and 2 M conditions. In contrast, the cells cultured with TGF-*β*1 exhibited a rounded shape, which reflected the synthesis of a thicker and more abundant matrix. In addition, Alcian blue staining and type II collagen immunolabeling (Figure 1(d)) showed that TGF-*β*1 was able to induce the significant synthesis of GAGs and type II collagen, which were distributed throughout the 3D-bioprinted substitutes. Additionally, ECM synthesis was more noticeable for the 1 M condition, as assessed by densitometry analysis (Figure 1(d)). We thus chose only the 1 M 3D bioprinting condition for the following experiments.

### 3.3. Longitudinal Study of the Combined Influences of Growth Factors and Hypoxia

#### 3.3.1. Mitochondrial Activity

**Figure 2:**
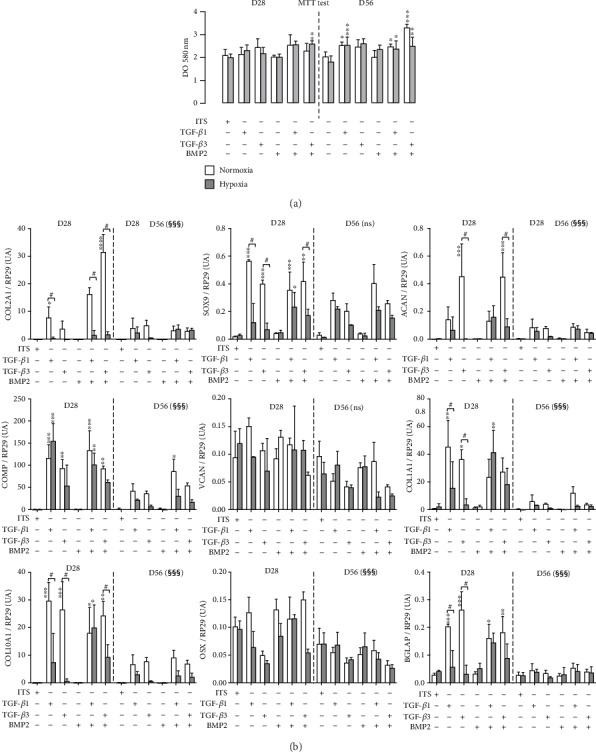
Influence on environmental factors in cell metabolism and gene expression in bioprinted cartilage substitutes on D28 and D56. (a) Influence of environmental factors on the MTT assay. In the first step, all comparisons were performed versus the control condition (ITS alone) for each growth factor and for each culture condition with one-way ANOVA followed by a Dunnett post hoc test. Data are presented as the mean ± SD. The experiment was carried out in triplicate. ^∗^*p* < 0.05; ^∗∗^*p* < 0.01; ^∗∗∗^*p* < 0.001 represents a significant difference versus ITS for each group. (b) Effect of environmental factors on gene expression. The expression of chondrogenic, hypertrophic, and fibrotic markers was investigated using real-time qPCR. In the first step, all comparisons were performed versus the respective control condition (ITS alone) for each growth factor and for each culture condition with 2-way ANOVA followed by Dunnett's post hoc test. Data are presented as the mean ± SD. ^∗^*p* < 0.05, ^∗∗^*p* < 0.01, and ^∗∗∗^*p* < 0.001 vs. ITS. Then, 3-way ANOVA was performed to assess the respective influences of time, growth factors, and normoxia/hypoxia. ^#^*p* < 0.05, ^##^*p* < 0.01, and ^###^*p* < 0.001, which means that hypoxia is significantly different than normoxia (Bonferroni's test). ^§§§^*p* < 0.001 means that there is a significant interaction between time (D28 vs. D56) and growth factors, meaning that gene expression was globally decreased at D56; ns: not significant. DO: absorbance.

On D28, neither growth factors nor hypoxia was shown to influence cell metabolism, with the exception of the TGF-*β*3/hypoxia combination, which was slightly stimulating (Figure 2(a)). On D56, TGF-*β*1 alone or in combination with BMP-2 induced a significant increase in mitochondrial activity, both in normoxia and hypoxia. Under hypoxia, TGF-*β*3 alone or in combination with BMP-2 significantly increased mitochondrial activity at D56. BMP-2 alone did not significantly influence mitochondrial activity. Finally, none of the experimental conditions was detrimental to cell metabolism compared with that of the respective control.

#### 3.3.2. Gene Expression

On D28, at this stage of differentiation, TGF-*β*1 and TGF-*β*3 induced the typical chondrogenic differentiation of MSCs within the 3D-bioprinted cartilage substitutes (Figure 2(b)). The significant overexpression of *COL2A1*, *COL10A1*, *ACAN*, *SOX9*, and *COMP* was observed, which supported the assumption that there was no difference between TGF-*β*1 and TGF-*β*3 treatment, except for *ACAN*, the expression of which was drastically increased in the presence of TGF-*β*3 only. Moreover, it is interesting to note that *OSX*, *BGLAP*, and *VCAN* gene expression remained negligible. BMP-2 alone was not able to induce any overexpression of the studied genes, but when it was combined with TGF-*β*1 or TGF-*β*3, BMP-2 significantly potentiated *COL2A1* expression (2-fold for TGF-*β*1+BMP-2 vs. TGF-*β*1 and 6-fold for TGF-*β*3+BMP-2 vs. TGF-*β*3). Globally, hypoxia repressed gene expression, and *COL2A1*, *ACAN*, and *COMP* expression was strongly decreased between D28 and D56, while the expression of *SOX9* (as a “chondromaster” gene) remained stable. In addition, the expression of hypertrophic and fibrogenic genes, i.e., *COL10A1* and *COL1A1*, was dramatically decreased under hypoxia between D28 and D56.

#### 3.3.3. Extracellular Matrix Production

**Figure 3:**
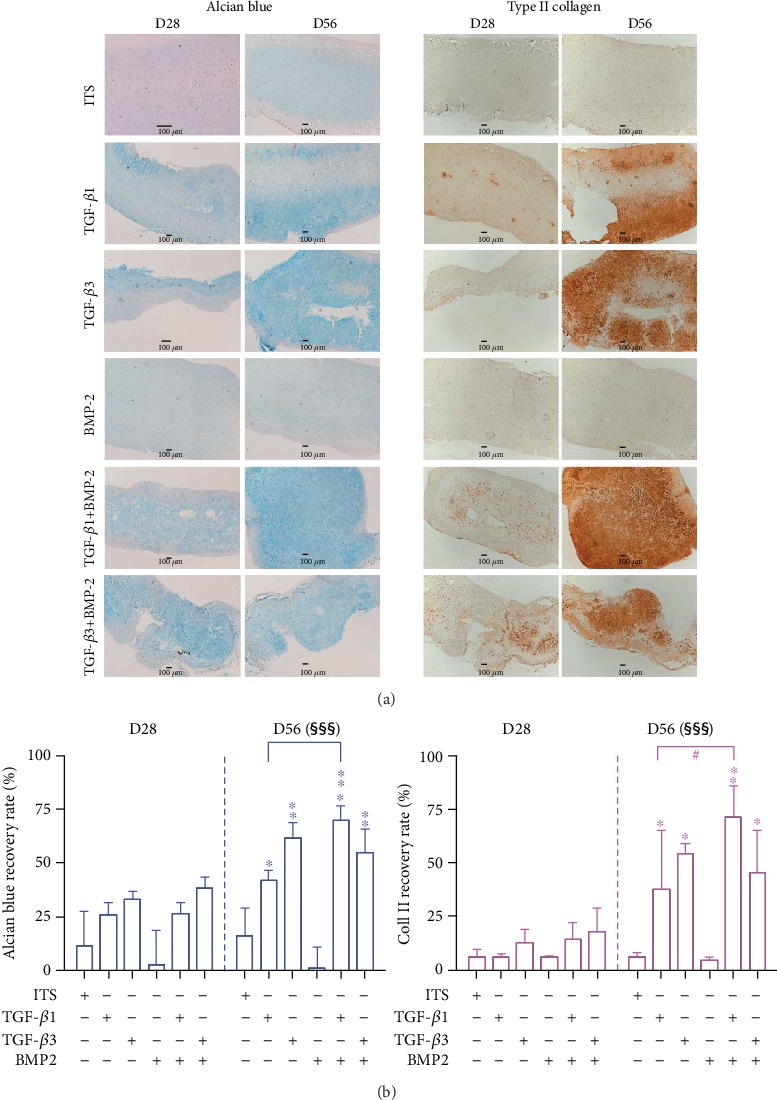
Histological and immunohistochemical analyses of 3D-bioprinted substitutes seeded with MSCs in various conditions under normoxia. (a) Proteoglycans were observed by Alcian blue staining, and type II collagen was observed using immunohistochemistry. Scale bars: 100 *μ*m (for the sake of clarity, hypoxia images have not been presented, as hypoxia had no significant influence on staining). (b) Quantitative analysis of histological images (scale bar: 100 *μ*m) with Alcian blue staining and immunohistochemical evidence of type II collagen in 3D-printed cartilage substitutes was performed with ImageJ. The results are expressed as the percentage mean ± SD of the positively stained area (4 to 6 images). In the first step, 1-way ANOVA followed by Dunnett's test was performed to assess the significance of the difference between each condition and its respective ITS control (normoxia and hypoxia) on D28 and D56. ^∗^*p* < 0.05, ^∗∗^*p* < 0.01, ^∗∗∗^*p* < 0.001; in the second step, the significant values were compared to the pertinent values with Bonferroni's test (^#^*p* < 0.05). Three-way ANOVA was performed to assess the respective effects of growth factors, hypoxia, and time (D28 and D56). There was a significant interaction between time and growth factors, meaning that staining was more marked on D56 than on D28 (^§§§^*p* < 0.001). In contrast, hypoxia did not have an effect at both staining intensities.

**Figure 4:**
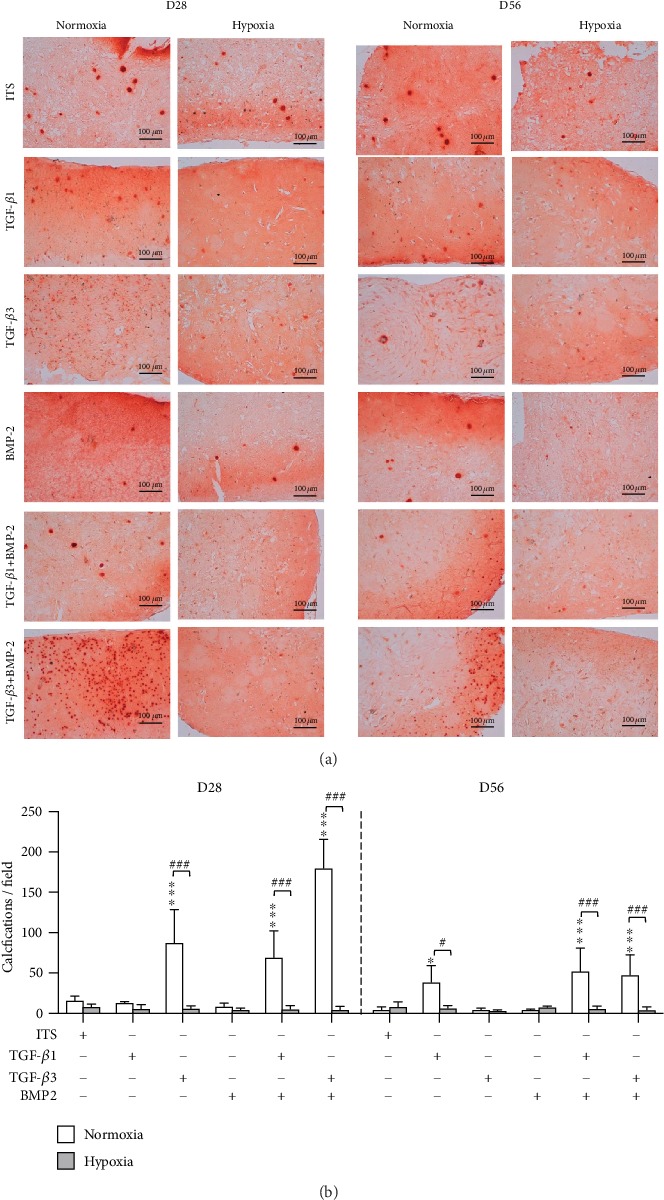
Histological analyses of 3D-bioprinted substitutes seeded with MSCs in various conditions under normoxia and hypoxia. (a) Microcalcifications were observed by alizarin red staining. Scale bars: 100 *μ*m. (b) Quantitative analysis of histological images (scale bar: 100 *μ*m) was performed with ImageJ. The results are expressed as the number of calcium deposits per field at 4x magnification (mean ± SD, 4-6 images per condition). In the first step, 1-way ANOVA followed by Dunnett's test was performed to assess the significance of the difference between each condition and its respective ITS control (normoxia and hypoxia) on D28 and D56. ^∗^*p* < 0.05, ^∗∗∗^*p* < 0.001; in the second step, 2-way ANOVA followed by Bonferroni's test was performed to assess the specific effect of hypoxia: ^#^*p* < 0.05, ^###^*p* < 0.001.

Under normoxia, a very low level of proteoglycan and type II collagen synthesis was observed at D28 and D56 in both ITS and BMP-2 culture conditions (Figure 3). In contrast, TGF-*β*1 and TGF-*β*3 alone or combined with BMP-2 induced significant and progressive proteoglycan and type II collagen accumulation. However, the TGF-*β*1 and BMP-2 combination was shown to induce higher GAG production when compared to that induced by TGF-*β*1 alone. Finally, hypoxia did not influence ECM production on either D28 or D56 (data not shown for the sake of clarity in Figure 3, see Suppl 5a and 5b) but significantly reduced the formation of calcium deposits in cartilage substitutes under normoxia conditions at D28 and D56 (Figure 4).

## 4. Discussion

In the present study, we confirmed the usefulness of the extrusion-based 3D bioprinting of composite bioink for the production of TGF-*β*-inducible MSC cartilage substitutes. We first established the lack of effects of both extrusion bioprinting and polymerization processes on MSC metabolism. Then, we validated the presence of a low MSC concentration (1 M), found in native healthy hyaline cartilage. This concentration was shown to lead to viable substitutes with activated chondrogenic genes under the influence of TGF-*β*1. In terms of the culture environment, hypoxia alone prevented the occurrence of calcifications and TGF-*β*1/3 combined with BMP-2 resulted in significantly enhanced chondrogenicity over that of TGF-*β*1 or TGF-*β*3 alone.

MSC three-dimensional (3D) bioprinting is an emerging technology that is expected to revolutionize the field of regenerative medicine [6, 20, 38–40], including hyaline articular cartilage engineering [41–52]. Previous tissue engineering approaches for cartilage repair usually failed to generate functional tissues recapitulating the zonal organization, extracellular matrix (ECM) content, and biomechanical properties of native cartilage. Various scaffolds and printers have been used for cartilage regeneration. For extrusion-based 3D bioprinting, the bioink viscoelasticity was shown to be a crucial factor for the cell survival rate when the bioprinting speed and extrusion flux were constant [53]. Furthermore, its composition represents one of the critical factors because of its direct influence on printability and onto cells in the designed cartilage substitutes. Our innovative bioink, previously designed for bioprinted skin [26], appears to be a good candidate for cartilage regenerative medicine.

Natural biological materials are easy to work with, are biodegradable without any waste, unlike polymers, and possess the advantage of chemical similarity with ECM components. In our experimental conditions, a composite bioink of alginate, gelatin, and fibrinogen allowed us to design *in vitro* cartilage substitutes and to obtain good quality synthesized ECM after 56 days of culture in the presence of a cocktail of TGF-*β*1 and BMP-2. Alginate and gelatin have been formerly used as 3D systems to promote the development of hyaline-like cartilage tissue obtained from the chondrogenic differentiation of MSCs with an ECM enriched in type II collagen and proteoglycans [54–57]. Native gelatin alone can hardly provide mechanical stability but is often used for bioprinting in combination with other biomaterials [56]. Daly et al. showed that alginate and agarose hydrogels supported the development of a more hyaline cartilage-like phenotype [58]. Additionally, fibrinogen is a soluble plasma protein that self-assembles into fibrin in the presence of thrombin and thus plays an important role in the stability of 3D-bioprinted shapes. With this in mind, fibrinogen was used in bioink to regulate cell differentiation and self-organization. At low concentrations (1–5 mg/mL), fibrinogen provides a suitable matrix for cell migration and differentiation. Conversely, fibrinogen concentrations exceeding 10 mg/mL induce a decrease in pore size. Furthermore, fibrin was shown to increase the stability of the matrix but to decrease F-actin organization [59]. This pilot study confirms the relevance of a universal ink for regenerative medicine of stratified collagen tissue. It has proven its worth in dermatology for artificial skin design, and its components are similar to those used for many years in cartilage tissue engineering.

Our results demonstrate that our custom-made extrusion-based 3D bioprinting method allows the layer-by-layer printing of 4 mm thick chondral substitutes that mimic the 3D environment necessary to induce and maintain MSC chondrogenic differentiation. Our bioprinting system uses a tronconical nozzle to ensure the best preservation of cell function, as previously demonstrated [60]. To this end, we generated nonprinted controls to assess the influence of shear stress on printed cells for 28 days. No effect of the 3D bioprinting process and polymerization was observed on cell viability. Typically, the influence of 3D bioextrusion depends on the stress during extrusion related to viscosity, the applied pressure, and the nozzle diameter. Our results are concordant with previous data demonstrating that bioprinting processes with extrusion, inkjet, or laser-based bioprinting technologies did not affect the viability of cells immediately or in the long term [61–66]. It is noteworthy that the cell viability was greater than 90% after the 3D bioprinting process and cross-linking of the bioink containing CaCl_2_ solution or thrombin [67, 68]. Conversely, cell viability could be affected by shear forces applied during the mixing of the bioink or during long postprinting cross-linking processes [69]. Currently, live/dead cell viability is often the only assay performed to evaluate the safety of a bioprinting process [53, 70]. In our experimental conditions, longitudinal real-time measurement of mitochondrial activity for up to 28 days, with or without TGF-*β*1, showed no alteration of the cells within the substitutes. Our composite bioink thus provided a biocompatible environment that preserved MSC metabolic activity and the ability to produce cartilaginous ECM (proteoglycans and type II collagen).

Based on previous studies demonstrating the chondrogenic differentiation of MSCs in hydrogels treated with growth factors such as TGF-*β*1 [71, 72], we confirmed the ability of 3D-bioprinted MSCs in an alginate-based bioink to produce a cartilaginous matrix with a low cell density. In the field of cartilage tissue engineering, most of the published studies use a high cell density, such as 10-20 million cells/mL, for chondrocytes [73, 74]. This is the same range used for MSC density [30, 48, 58, 74–77] to attain acceptable ECM production. In contrast, studies using 1-3 million cells/mL are rare [23, 78, 79]. In the present study, using this cell concentration, the chondrogenic cell metabolism and gene expression on D28 were consistent with the results of our previous works using alginate/hyaluronate hydrogels [80] or collagen sponges [81] under normoxia with or without TGF-*β*1 exposure. Additionally, the low cellular concentration individualizes the cells into each other following the structure of the cartilage, where cell communication is mainly paracrine. This low cell concentration is in agreement with the small percentage of cells in hyaline cartilage, especially in the intermediate zone. Additionally, in regenerative medicine, the low number of cells is an advantage. It facilitates nutrient diffusion and minimizes the gradients of ECM production observed with high cell concentration [27, 82, 83]. A low density makes it easier to use the patient's samples and to obtain the number of cells sufficient to produce substitutes quickly. It also reduces the time of cellular expansion to avoid marker modification and to reduce the risk of contamination in clinical practice.

In the 3D-bioprinted cartilage substitutes, we observed a significant increase in *COL2A1*, *ACAN*, and *SOX9*. Globally, on D28, hypoxia only repressed chondrogenic and osteogenic/hypertrophic gene expression during the differentiation phase. During the ECM synthesis period (D56), the expression of all genes was decreased, except that of *SOX9*, which was likely because of its role in collagen synthesis. In other published studies, this trend was not observed for *ACAN* and *COL2A1* for bioprinted chondrocytes [7]. In contrast, and as expected, ECM synthesis was more pronounced on D56 and the densitometry measurements after ECM staining confirmed the benefit of combining TGF-*β*1 and BMP-2 [75, 84–87]. As we previously reported in a study of synovial fluid MSCs, we observed no influence of BMP-2 alone on both gene and protein levels [37]. Unfortunately, the phenotype of MSCs in cartilage repair is unstable so that differentiation continues along the endochondral ossification pathway. In other words, when pushed towards chondrogenesis, MSCs tend to evolve into the hypertrophic/osteogenic commitment. Hypertrophy is marked by cell volume increase and ECM remodeling. These changes are regulated by the transcription factors Runx2/MEF2C, which regulate transcription of collagen X. The major effect of hypoxia was to prevent the occurrence of intra-ECM microcalcifications [88], which are characteristic of the phenotypic drift towards an osteoblast phenotype, as demonstrated herein with alizarin red staining. As extensively reported by Pattappa et al. [89], *COL10A1* and *BGLAP* expression was strongly inhibited on D28 under hypoxia (or chondroxia/physioxia), thus preventing hypertrophy and ancillary ECM calcification/ossification [89–92].

## 5. Conclusions

The present study demonstrated a promising approach for articular cartilage engineering by using an extrusion-based 3D bioprinting process and a low concentration (1 M) of human bone MSCs. Our innovative bioink combining alginate, gelatin, and fibrinogen is safe for MSCs and allows the generation of TGF-*β*-inducible engineered cartilage substitutes. In this first step experiment, we reproduced the structure of the chondral intermediate zone, with round cells in an abundant MEC within the four layers of 1 mm. 4 mm is comparable to human cartilage thickness with paracrine communication between chondrocytes. ACAN and COL2A were present. No vessel was detected. Under such conditions, hypoxia did not significantly improve ECM synthesis but prevented calcium deposition. Finally, hypoxia stabilized the chondrogenic phenotype, especially when using a combination of TGF-*β*1 and BMP-2. These results require further in vitro biomechanical studies and *in vivo* studies to confirm the biocompatibility/biofunctionality and the biointegration of these cartilage substitutes in ectopic [93] and orthotopic [79] conditions. One next step will be to vary MSC origin (e.g., bone, synovial MSCs) and environment (hyaluronate, chondroitin sulfate, collagen, and hydroxyapatite) in each layer to reproduce the superficial, middle, deep, and calcified zones.

## Figures and Tables

**Table 1:** Quantitative real-time PCR primers.

Gene	Primer sequences		Annealing temperature (°C)	Amplicon size (bp)	Accession number
*RP29*	Fwd Rev	5′-AGATGGGTCACCAGCAGCTGTACTG-3′ 5′-AGACACGACAAGAGCGAGAA-3′	60	73	NM_001032
*COL2A1*	Fwd Rev	5′-ATGACAATCTGGCTCCCAAC-3′ 5′-GAACCTGCTATTGCCCTCTG-3′	55	200	NM_001844
*SOX9*	Fwd Rev	5′-GAGCAGACGCACATCTC-3′ 5′-CCTGGGATTGCCCCGA-3′	55	118	NM_000346
*ACAN* (*aggrecan*)	Fwd Rev	5′-TCGAGGACAGCGAGGCC-3′ 5′-TCGAGGGTGTAGCGTGTAGAGA-3′	63	85	NM_001135
*COMP*	Fwd Rev	5′-ACAATGACGGAGTCCCTGAC-3′ 5′-TCTGCATCAAAGTCGTCCTG-3′	60	115	NM_000095
*VCAN* (*versican*)	Fwd Rev	5′-TGTTCCTCCCACTACCCTTG-3′ 5′-CTTCCACAGTGGGTGGTCTT-3′	62	122	NM_001164098
*COL1A1*	Fwd Rev	5′-AGGTGCTGATGGCTCTCCT-3′ 5′-GGACCACTTTCACCCTTGT-3′	60	104	NM_000088
*COL10A1*	Fwd Rev	5′-GCTAAGGGTGAAAGGGGTTC-3′ 5′-CTCCAGGATCACCTTTTGGA-3′	60	118	NM_000493
*OSX* (*osterix*)	Fwd Rev	5′-CCCCACCTCTTGCAACCA-3′ 5′-GGCTCCACCACTCCCTTCTAG-3′	60	102	NM_152860
*BGLAP* (*osteocalcin*)	Fwd Rev	5′-GTGCAGAGTCCAGCAAAGGT-3′ 5′-TCAGCCAACTCGTCACAGTC-3′	62	175	NM_199173

## Data Availability

The datasets taken during and/or analyzed during the current study are available from the corresponding author on reasonable request.

## Abbreviations

1 M, 2 M: One or 2 million cells/mL in bioink
3D: Three-dimensional
*ACAN*: Aggrecan (gene)
ANOVA: Analysis of variance
bFGF: Basic fibroblast growth factor
*BGLAP*: Osteocalcin (gene)
BMP-2: Bone morphogenetic protein 2
CO_2_: Carbon dioxide
*COL10A1*: Collagen type X alpha 1 chain (gene)
*COL2A*: Collagen type II alpha 1 chain (gene)
*COMP*: Cartilage oligomeric matrix protein (gene)
DMEM: Dulbecco's modified Eagle medium
EBB: Extrusion-based bioprinting
DO: Absorbance
ECM: Extracellular matrix
FBS: Fetal bovine serum
GAG: Glycosaminoglycan
HES: Hematoxylin erythrosine saffron
ITS: Insulin-transferrin-selenium
M: Million
MSCs: Mesenchymal stem cells
MTT: 3(4,5-Dimethylthiazol-2-yl)-2,5-diphenyltetrazolium bromide
OA: Osteoarthritis
*OSX*: Osterix (gene)
PBS: Phosphate-buffered saline
qRT-PCR: Quantitative reverse transcription polymerase chain reaction
RNA: Ribonucleic acid
*RPS29*: Ribosomal protein S29 (gene)
*SOX9*: Sex-determining region-related HMG-box9 (gene)
TGF-*β*: Transforming growth factor beta [1 or 3]
*VCAN*: Versican (gene).

## Supplementary Materials

Supplementary Materials Supplementary Data 1: general design of the study. Supplementary Data 2: bioprinting process. Supplementary data 3: DNA assay (Hoechst's method) on D3. Supplementary data 4: HES staining analyses of 3D-printed substitutes seeded with MSCs (1 M or 2 M). Supplementary data 5a: histological analyses (Alcian blue) of 3D-bioprinted substitutes seeded with MSCs in various conditions under normoxia and hypoxia. Supplementary data 5b: immunohistochemical analyses (type II collagen) of 3D-bioprinted substitutes seeded with MSCs in various conditions under normoxia and hypoxia. Supplementary data 5c: histological analyses (HES) of 3D-bioprinted substitutes seeded with MSCs in various conditions under normoxia and hypoxia.
