# Supplementary material for: Combining Innovative Bioink and Low Cell Density for the Production of 3D-Bioprinted Cartilage Substitutes: A Pilot Study
Source: Stem Cells Int. 2020 Jan 21;2020:2487072. doi: 10.1155/2020/2487072 (PMC7201838; doi:10.1155/2020/2487072)
Supplement: Supplementary Materials — Supplementary Data 1: general design of the study. Supplementary Data 2: bioprinting process. Supplementary data 3: DNA assay (Hoechst's method) on D3. Supplementary data 4: HES staining analyses of 3D-printed substitutes seeded with MSCs (1 M or 2 M). Supplementary data 5a: histological analyses (Alcian blue) of 3D-bioprinted substitutes seeded with MSCs in various conditions under normoxia and hypoxia. Supplementary data 5b: immunohistochemical analyses (type II collagen) of 3D-bioprinted substitutes seeded with MSCs in various conditions under normoxia and hypoxia. Supplementary data 5c: histological analyses (HES) of 3D-bioprinted substitutes seeded with MSCs in various conditions under normoxia and hypoxia. [file 2487072.f1.pdf]

8. Supplementary data

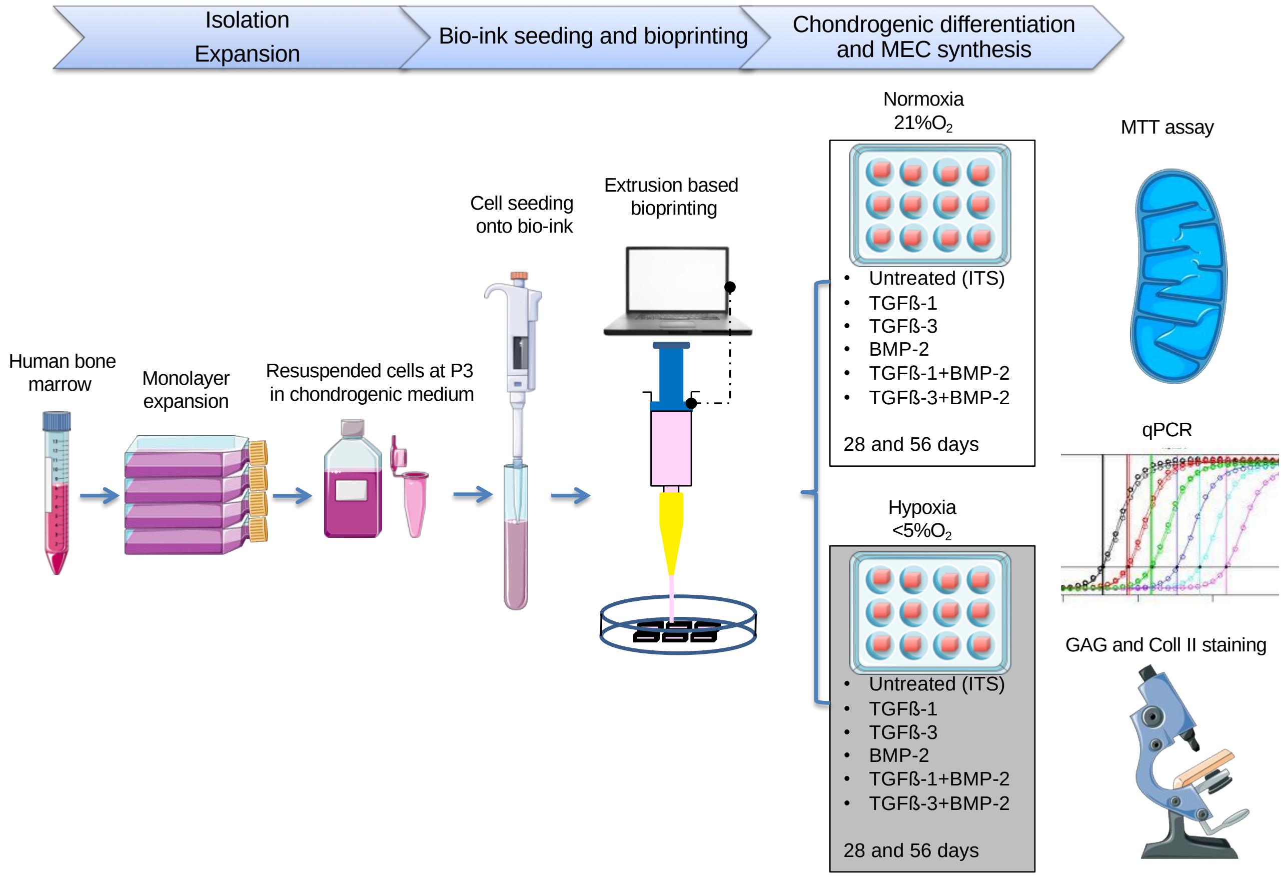

Supplementary Data 1: General design of the study

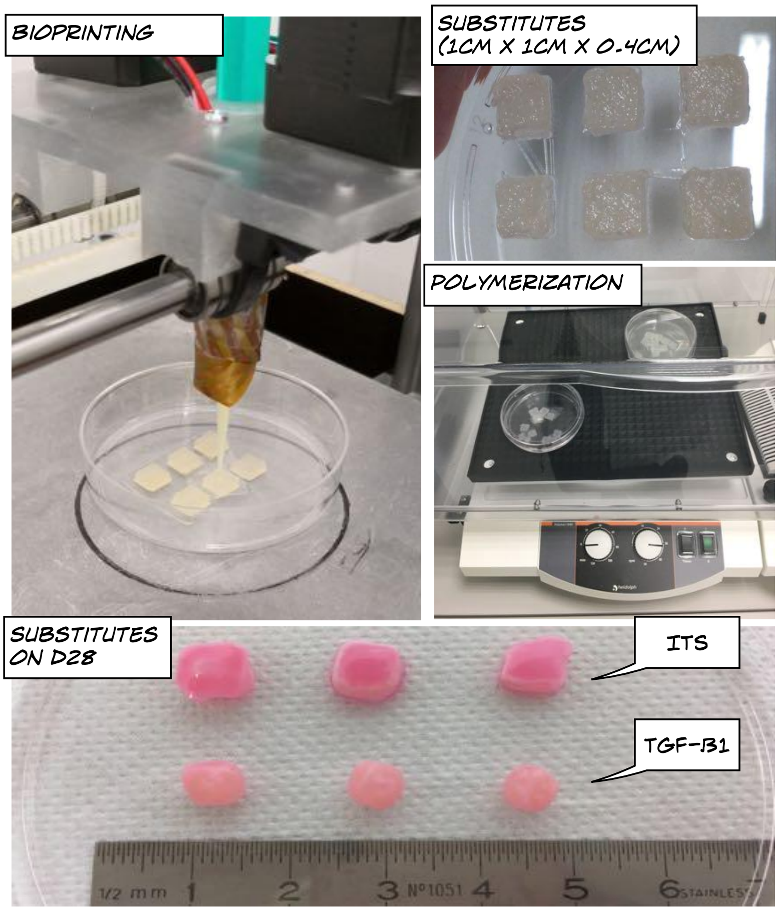

Supplementary Data 2: Bioprinting process

All solutions (fibrinogen, alginate and gelatin) are prepared in sterile conditions the day before printing. They are placed at 37°C for a good dissolution of the powders. Gelatin is obtained in NaCl (20%). Fibrinogen is prepared in culture medium (160 mg / 2mL) and alginate is prepared in NaCl (4%). The cells are taken up in 2 mL of fibrinogen to which 4 mL of gelatin and 2 mL of alginate are added (total of 8 mL). The cells were trypsinized, counted, and resuspended in 2 mL of fibrinogen solution (160 mg in 2 mL culture medium). Four mL of gelatin (20% in NaCl) and 2 mL of alginate (4% in NaCl) are added. The 8 mL obtained in this way are homogenized using a Microman® "special viscous media" pipette, then taken up in a 10 mL syringe. The syringe containing the bio-ink is then maintained at room temperature for 30 minutes, which is the time required to obtain a bio-ink whose viscosity is compatible with good printability. This 30-minute time was previously developed by Pourchet et al. in her previous work. At the end of printing, the gels are gently recovered with a sterile flat spatula and placed in another Petri dish containing 400 µL of a thrombin solution (500 U/mL) in 20 mL CaCl2 (3% in NaCl) to allow the bio-gels to polymerize. According to the preliminary developments carried out by C MARQUETTE, allowing a proper maintenance of the bio-gels and good viability of the cells, we carried out polymerization of 1 hour under agitation at 37°C. At the end of this polymerization time, a 5-minute wash with PBS is carried out, and then each bio-gel is individually placed in a 6-well culture plate (1 bio-gel per well) to be cultured under the different conditions. Scale is graded in cm.

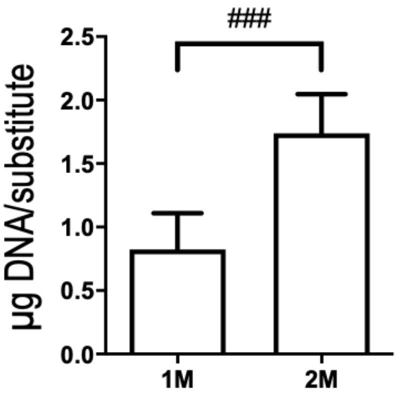

Supplementary data 3:
DNA assay (Hoechst's method) showing a 2-fold greater amount of DNA at a density of 2 M compared to that at 1 M on D3. Data are mean ± SD. ### : p<.001, Student's t test

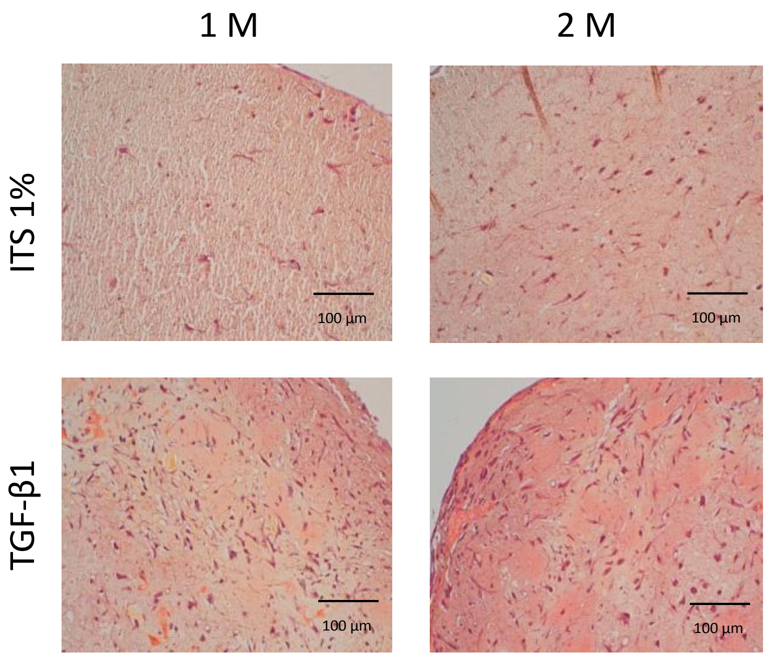

Supplementary data 4:
HES staining analyses of 3D-printed substitutes seeded with MSCs (1 M or 2 M) in both culture conditions (1% ITS or TGF-β1) on D28. Bar: 100 µm. As expected, TGF-ß1 increased cell density in substitutes. Nuclei are clearly individualized. No sign of cell death is depicted.

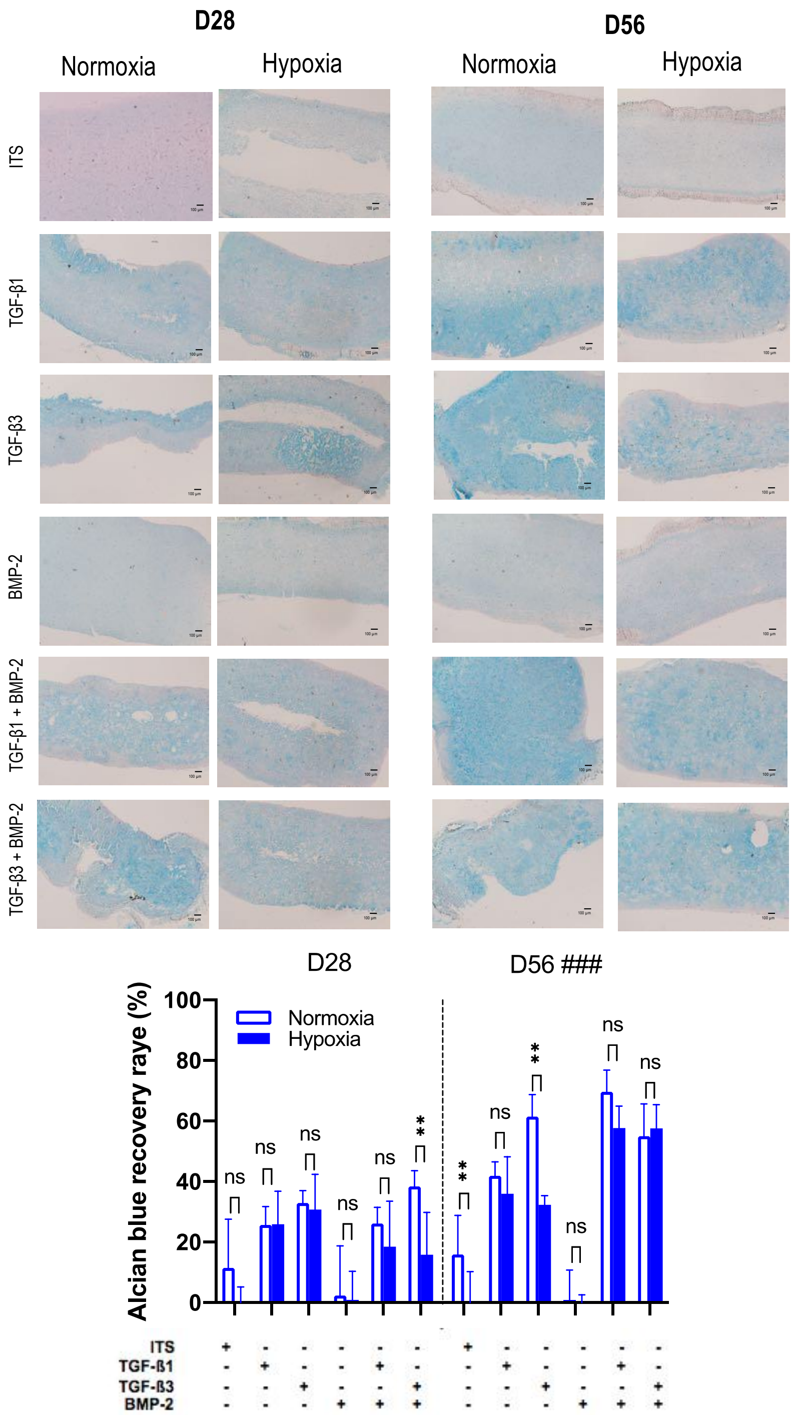

Supplementary data 5a. Histological analyses of 3D-bioprinted substitutes seeded with MSCs in various conditions under normoxia and hypoxia.

Proteoglycans were observed by Alcian blue staining. Scale bars: 100 µm.

Quantitative analysis (bottom) of histological images with Alcian blue staining in 3D-printed cartilage substitutes was performed with ImageJ. The results are expressed as the percentage mean ± SD of the positively stained area (4 to 6 images). Three-way ANOVA was performed to assess the respective effects of growth factors, hypoxia and time (D28 and D56). There was a significant interaction (between time and growth factors, meaning that staining was more marked on D56 than on D28. ### p<0.001In contrast, hypoxia did not have a global significant positive interaction on D28 and D56 on Alcian blue staining. **p>0.01 Dunnett's test

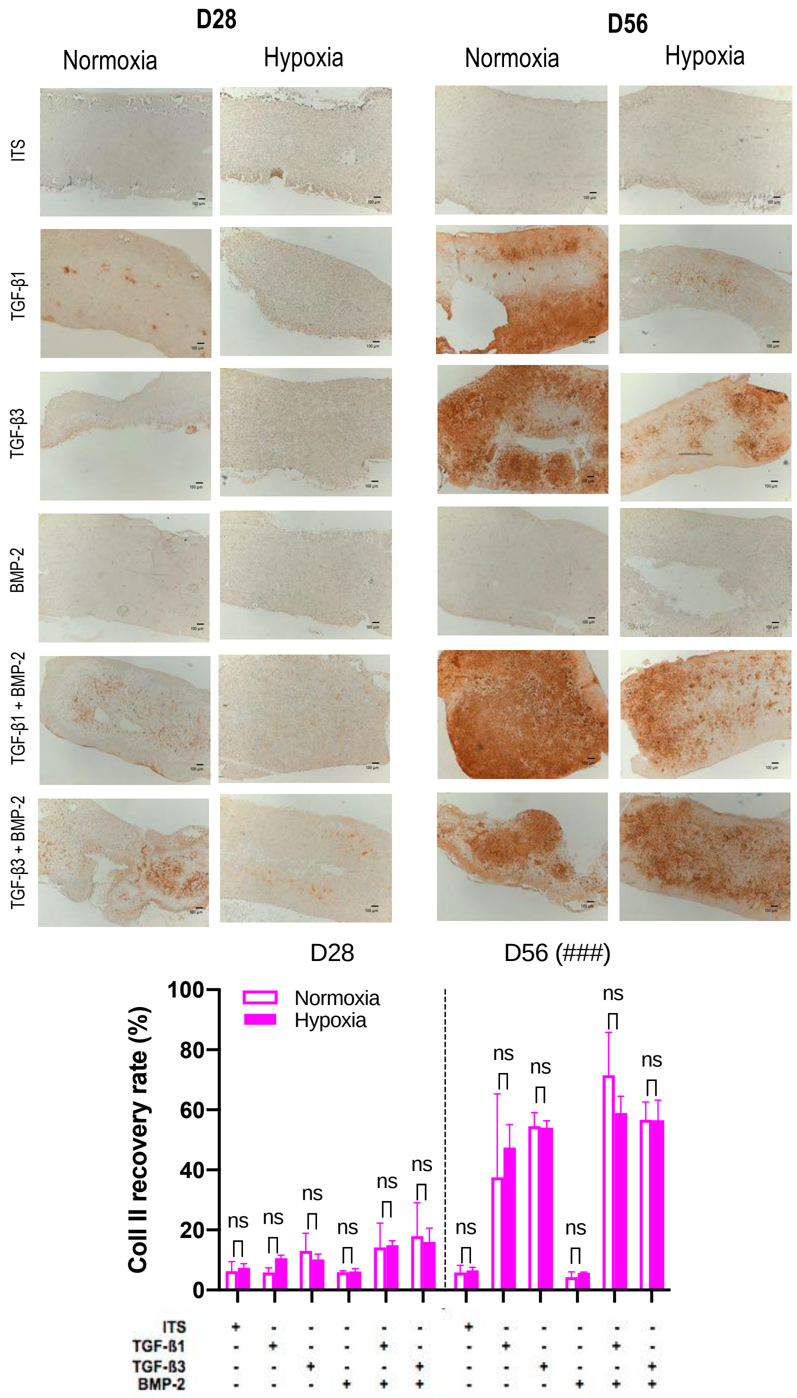

Supplementary data 5b. Immunohistochemical analyses of 3D-bioprinted substitutes seeded with MSCs in various conditions under normoxia.

Type II collagen was observed using immunohistochemistry. Scale bars: 100 µm.

Quantitative analysis (bottom) of histological images (scale bar: 100 µm) with immunohistochemical evidence of type II collagen in 3D-printed cartilage substitutes was performed with ImageJ. The results are expressed as the percentage mean ± SD of the positively stained area (4 to 6 images). Three-way ANOVA was performed to assess the respective effects of growth factors, hypoxia and time (D28 and D56). There was a significant interaction between time and growth factors, meaning that staining was more marked on D56 than on D28 ($p<0.001$, ###). In contrast, hypoxia did not have an effect on staining intensity on D28 and D56.

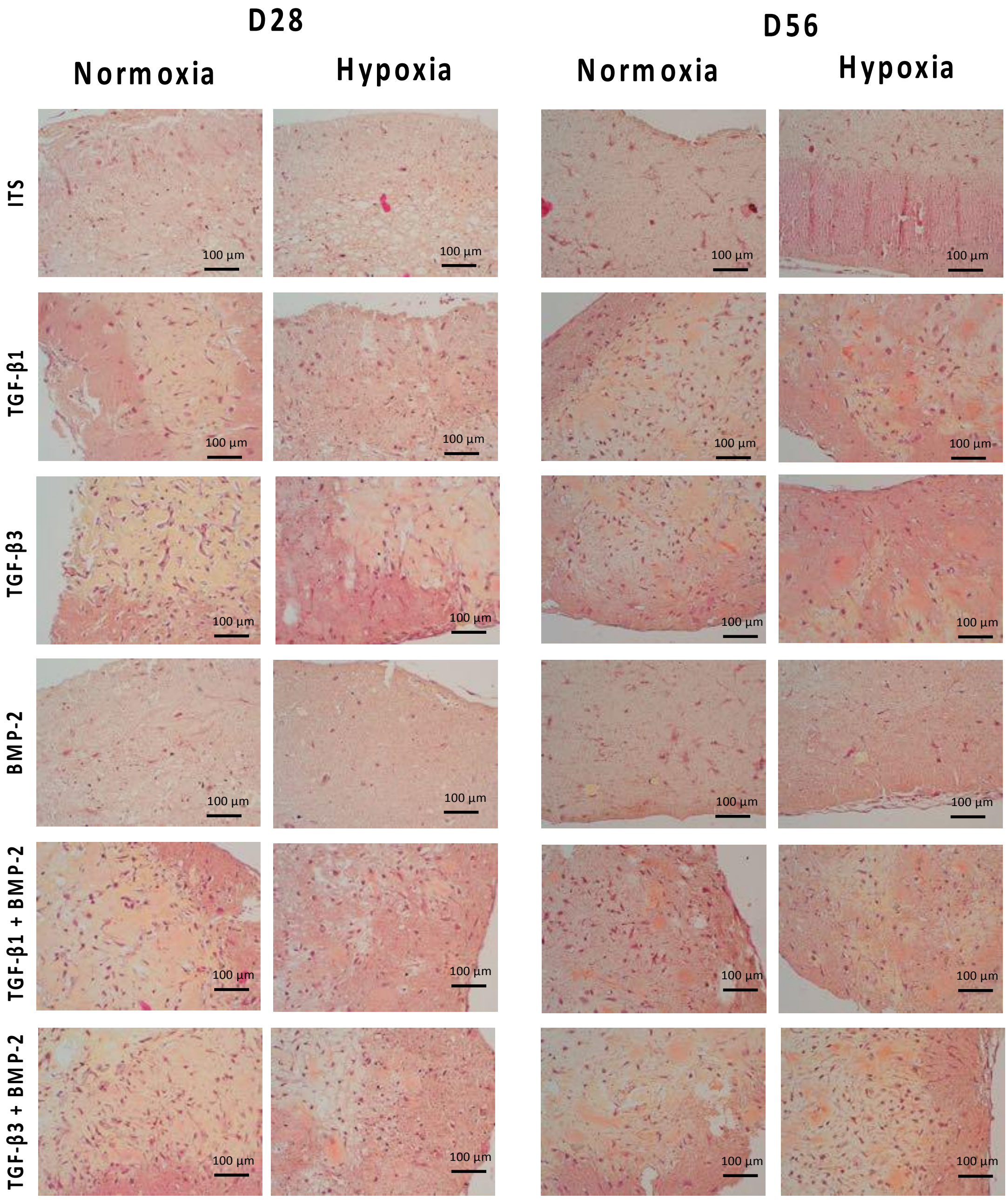

Supplementary data 5c. Histological analyses of 3D-bioprinted substitutes seeded with MSCs in various conditions under normoxia and hypoxia.

Cell morphology and viabilitywas observed using HES. Scale bars: 100 µm.

Growth factor TGF-ß1increased cell density. Hypoxia did not affect cell viability.

The cells are homogeneously distributed in the extracellular matrix.
